# Activity of afatinib in patients with NSCLC harboring novel uncommon *EGFR* mutations with or without co-mutations: a case report

**DOI:** 10.3389/fonc.2024.1347742

**Published:** 2024-05-06

**Authors:** Petros Christopoulos, Franziska Herster, Petra Hoffknecht, Markus Falk, Markus Tiemann, Hans-Georg Kopp, Andre Althoff, Anja Stammberger, Eckart Laack

**Affiliations:** ^1^ Department of Oncology, Thoraxklinik and National Center for Tumor Diseases at Heidelberg University Hospital, Heidelberg, Germany; ^2^ Thoracic Oncology, Translational Lung Research Heidelberg, Member of the German Center for Lung Research (DZL), Heidelberg, Germany; ^3^ Robert Bosch Center for Tumor Diseases (RBCT), Robert Bosch Hospital, Stuttgart, Germany; ^4^ Lungenzentrum Osnabrueck, Franziskus-Hospital Harderberg, Georgsmarienhütte, Germany; ^5^ Lung Cancer Network NOWEL.org, Oldenburg, Germany; ^6^ Molecular Pathology, Institute of Hematopathology Hamburg, Hamburg, Germany; ^7^ Department of Pulmonology, Thoraxzentrum Offenbach, Sana Klinikum Offenbach, Offenbach, Germany; ^8^ Oncology, Boehringer Ingelheim Pharma GmbH & Co. KG, Ingelheim, Germany; ^9^ Hemato-Oncology Hamburg, Hamburg, Germany

**Keywords:** EGFR, non-small cell lung cancer (NSCLC), afatinib, uncommon mutation, tyrosine kinase inhibitor

## Abstract

Epidermal growth factor receptor (EGFR) tyrosine kinase inhibitors (TKIs) represent first-line standard of care in unresectable *EGFR* mutation-positive (*EGFR*m+) non-small cell lung cancer (NSCLC). However, 10–20% of patients with *EGFR*m+ NSCLC have uncommon *EGFR* variants, defined as mutations other than L858R substitutions or exon 19 deletions. NSCLC harboring uncommon *EGFR* mutations may demonstrate lower sensitivity to targeted agents than NSCLC with L858R or exon 19 deletion mutations. Prospective clinical trial data in patients with NSCLC uncommon *EGFR* mutations are lacking. Afatinib is a second-generation TKI and the only Food and Drug Administration-approved drug for some of the more prevalent uncommon *EGFR* mutations. We present a series of seven case reports describing clinical outcomes in afatinib-treated patients with NSCLC harboring a diverse range of extremely rare mutations with or without co-mutations affecting other genes. *EGFR* alterations included compound mutations, P-loop αC-helix compressing mutations, and novel substitution mutations. We also present a case with NSCLC harboring a novel *EGFR*::*CCDC6* gene fusion. Overall, the patients responded well to afatinib, including radiologic partial responses in six patients during treatment. Responses were durable for three patients. The cases presented are in line with a growing body of clinical and preclinical evidence that indicating that NSCLC with various uncommon *EGFR* mutations, with or without co-mutations, may be sensitive to afatinib.

## Introduction

Activating epidermal growth factor receptor (*EGFR)* mutations occur in 14–38% of non-small cell lung cancer (NSCLC) (1). EGFR tyrosine kinase inhibitors (TKIs) represent first-line standard of care in unresectable *EGFR* mutation-positive (*EGFR*m+) disease (2). Most *EGFR*m+ NSCLC is driven by the so-called classical or common *EGFR* mutations: L858R or exon 19 deletions (Del19) (3). Approximately 10–20% of *EGFR*m+ NSCLC cases harbor uncommon *EGFR* mutations, defined as activating *EGFR* mutations other than L858R and Del19 (3–5). Different variants demonstrate varying sensitivity to EGFR TKIs, and uncommon *EGFR* mutations show lower sensitivity to many targeted agents than classical *EGFR* mutations; therefore, precise characterization of uncommon *EGFR* mutations is important to optimize treatment strategies (4).

The most prevalent uncommon *EGFR* variants in NSCLC are S768I, L861Q, and G719X, for which the preferred first-line treatments in advanced disease are afatinib or osimertinib (6–9). Afatinib is the only U.S. Food and Drug Administration-approved drug against S768I, L861Q, and G719X *EGFR* mutations with demonstrated efficacy in prospective clinical studies (10). Little prospective data exist for uncommon mutations; however, retrospective studies (7, 11–15) and databases (11, 16–18) provide some insight. Novel mutations continue to be identified that have no available clinical data to guide treatment decisions.

A recent preclinical investigation defined a structure-based classification system that permitted prediction of sensitivity to different generations of EGFR TKIs (first generation: erlotinib, gefitinib; second generation: afatinib, dacomitinib; third generation: osimertinib) (4). “Classical-like” mutations (e.g. L858R; Del19; S720P; L861Q/R) were predicted to be sensitive to all generations of EGFR TKI; “T790M-like” mutations (e.g. T790M; certain T790M-containing compound mutations) were predicted to be sensitive to third-generation EGFR TKIs; exon 20 insertions (e.g. S768dupSVD; A767dupASV) were predicted to be sensitive to exon 20 insertion-targeted compounds and second-generation EGFR TKIs; and P-loop αC-helix compressing (PACC) mutations (e.g. G719X; S768I; delE709_T710insD, and other uncommon *EGFR* mutations were predicted to be particularly sensitive to second-generation EGFR TKIs (4). PACC mutations occur across exons 18–21 and alter the orientation of the P-loop or αC-helix of EGFR, affecting interactions with certain TKIs. Second-generation TKIs do not interact with the P-loop of EGFR and are therefore predicted to have greater activity against PACC mutations than other generations of EGFR TKI (4). Some retrospective data support this prediction (4).

Treatment decisions can be very challenging in patients with NSCLC with multiple *EGFR* mutations (compound mutation) or uncommon *EGFR* mutations co-occurring with other gene alterations in the tumor. Treatment might be dependent on which mutation has the higher allele frequency (19) or which other cancer-related genes have co-occurring mutations (20, 21). For example, *TP53*, the most commonly mutated gene in NSCLC, co-occurs in ~65% of cases of *EGFR*m+ NSCLC, and has been associated with poor prognosis and primary/acquired resistance to EGFR TKIs (22–27).

This case series describes outcomes of patients with NSCLC harboring uncommon *EGFR* mutations who received afatinib. Cases were collected during routine clinical treatment across six centers in Germany between 2017 and 2023.

## Case descriptions

### Patients with an *EGFR* PACC mutation as part of a compound mutation

#### Case 1: G719A/L833F

**Figure 1:**
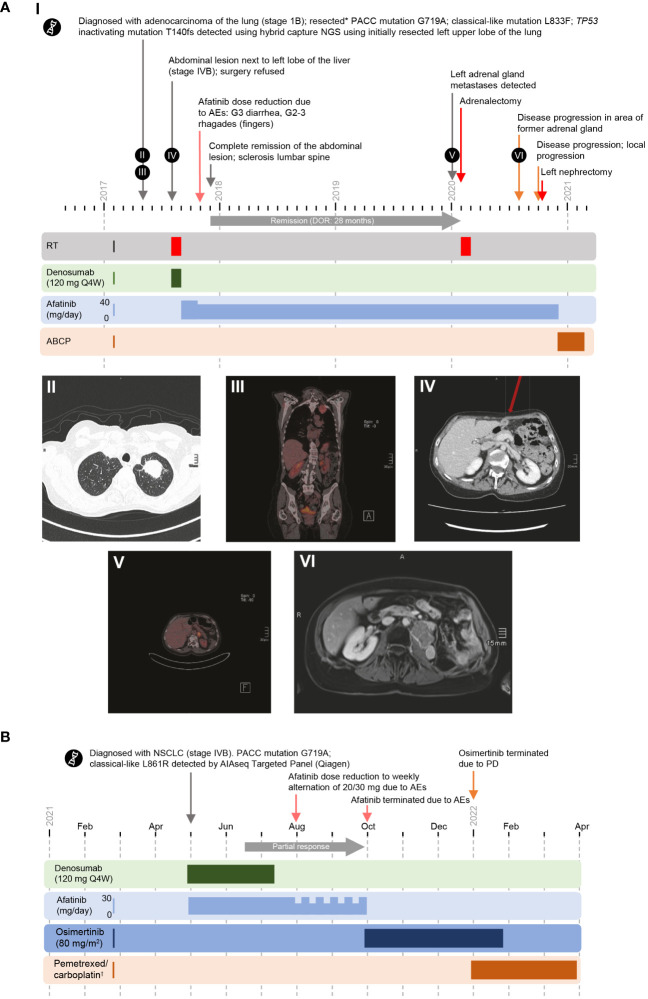
Cases 1 and 2 (PACC/classical compound *EGFR* mutation). **(A)** I Timeline of Case 1. II, III. May 2017. Tumor staging pT2a pN0 M0, St. IIA UICC 8. IV. August 2017. A single symptomatic metastasis and infiltration of the major psoas was detected. V. January 2020. Adrenal metastases detected. VI. August 2020. Disease progression was observed in the area of the former adrenal gland. **(B)** Timeline of Case 2. *Single symptomatic metastasis and infiltration of the major psoas. ^†^488.12 mg carboplatin AUC 80%; 796 mg pemetrexed 80%. ABCP, atezolizumab plus bevacizumab plus carboplatin plus paclitaxel; AE, adverse event; DOR, duration of response; G, grade; NGS, next generation sequencing; NSCLC, non-small cell lung cancer; PACC, P-loop and αC-helix compressing; PD, progressive disease; Q4W, every 4 weeks; RT, radiotherapy; UICC, Union for International Cancer Control.

After presenting with a cough, a 58-year-old female with a history of paroxysmal atrial fibrillation and hemangioma was diagnosed with lung adenocarcinoma (stage IB) in May 2017, and underwent resection of the left upper lobe and lymphadenectomy. In August 2017, a single symptomatic metastasis to the third lumbar spine vertebrae with infiltration of the major psoas was detected. The patient received radiotherapy and began treatment with denosumab (120 mg every 4 weeks; **Figure 1A**). However, in September 2017, a new abdominal lesion next to the left lobe of the liver was reported. The patient refused biopsy to confirm diagnosis of distant metastases. Hybrid capture next-generation sequencing (NGS) of the initially resected tumor tissue identified a novel compound *EGFR* mutation, comprising two substitution mutations on exons 18 (G719A, a PACC mutation) and 21 (L833F, a classical-like mutation). A *TP53* point inactivating mutation (p.T140 frame shift [non-activating mutation]) was also detected.

The patient began treatment with first-line afatinib, 40 mg once per day (QD), in September 2017. In November 2017, following grade 3 diarrhea, grade 2–3 stomatitis, and rhagades of the fingers, the dose of afatinib was reduced to 30 mg QD.

The patient achieved complete remission of the abdominal lesion, with the response lasting 28 months. Metastases were detected in the left adrenal gland in January 2020. In February 2020, the patient underwent adrenalectomy (R1) followed by radiotherapy and continued afatinib treatment. Disease was stable until June 2020.

In August 2020, disease progression was observed in the area of the former adrenal gland. Urinary retention was treated with a double J-tumor stent and the patient experienced urosepsis (*Proteus mirabilis*, two events) and nephroptosis. Following local progression and subsequent left nephrectomy in October 2020, afatinib therapy was terminated in December 2020, and the patient received second-line therapy with carboplatin/paclitaxel, atezolizumab, and bevacizumab. The total duration of afatinib treatment was 35 months.

#### Case 2: G719A/L861R

A 71-year-old male with a history of polymyalgia rheumatica consulted his general practitioner with concerns relating to a family history of cancer. In May 2021, elevated serum tumor markers were reported, and the patient was subsequently diagnosed with NSCLC (stage IVB; programmed death-ligand 1 [PD-L1]: 1%; pulmonary and bone metastases) (**Figure 1B**). NGS (QIAseq Custom Lung Panel, Qiagen) identified a novel compound mutation comprising substitution mutations on exon 18 (G719A, PACC) and exon 21 (L861R, classical-like).

The patient began treatment in May 2021, with first-line afatinib (30 mg QD) plus denosumab (120 mg every 4 weeks). Following presentation with exanthem (June/July 2021, treated with topical corticosteroid) and diarrhea (August 2021, treated with loperamide), the dose of afatinib was reduced to weekly alternation of 20/30 mg.

Partial responses (PRs) were reported in June, July, and August 2021. After approximately 5 months on treatment, stable disease (SD) was reported. However, afatinib was terminated in October 2021 owing to intolerable adverse events (AEs), and osimertinib (80 mg QD) was initiated. Progressive disease (PD) was reported in January 2022, which resulted in discontinuation of osimertinib and initiation of pemetrexed and carboplatin treatment.

### Patients with PACC exon 18 deletion insertion mutations

#### Case 3: delE709_T710insN

**Figure 2:**
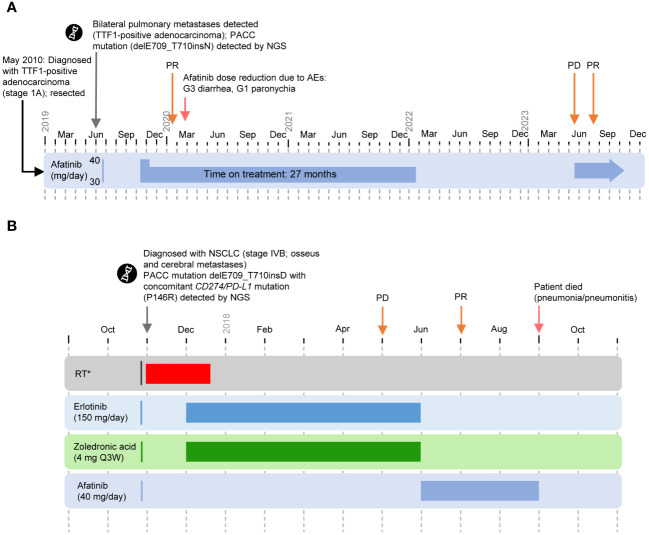
Cases 3 and 4. (PACC exon 18 deletion insertion mutation) **(A)** Timeline of Case 3. Afatinib was discontinued in January 2022 at the patient’s request. In May 2023, progression of the lung metastases was observed following a CT scan. Afatinib was resumed and PR was observed in July 2023. As of September 2023, the patient remains symptom free. **(B)** Timeline of Case 4. *Nov 2017: palliative RT (thoracic spine, lumbar, Os pubis left [5 Gy/Fraction]. WBRT: Nov–Dec 2017. Zoledronic acid: Dec 2017–Jun 2018. First-line treatment with erlotinib: Dec 2017–Jun 2018. Second-line treatment with afatinib: Jun–Sep 2018. AE, adverse event; G, grade; NGS, next-generation sequencing; NSCLC, non-small cell lung cancer; PACC, P-loop and αC-helix compressing; PD, progressive disease; PR, partial response; Q3W, every 3 weeks; RT, radiotherapy; TTF-1, thyroid transcription factor-1; WBRT, whole brain radiotherapy.

A 64-year-old male presented with persistent cough. A computerized tomography (CT) scan revealed pulmonary nodules on both sides of the lung, and following a biopsy by bronchoscopy the patient was diagnosed with thyroid transcription factor-1 (TTF1)-positive adenocarcinoma (stage IA) in May 2010, with resection in the same month (**Figure 2A**). Relevant comorbidities included arterial hypertension, treated with candesartan (32 mg). In June 2019, aged 73 years, he was diagnosed with bilateral pulmonary metastases (TTF1-positive adenocarcinoma), following biopsy by bronchoscopy. NGS detected an uncommon *EGFR* exon 18 deletion insertion mutation (delE709_T710insN), classified as PACC (4).

The patient began first-line afatinib (30 mg QD) in October 2019. Following emergence of grade 3 diarrhea, afatinib was paused and the patient was treated with loperamide and hydration until symptoms had resolved. Following grade 1 paronychia, the dose of afatinib was reduced to 20 mg QD. No further AEs occurred.

The patient achieved a best response of PR after 3 months of treatment with afatinib. The patient reported good quality of life with no clinical symptoms of disease. Afatinib was continued for 27 months and was discontinued in January 2022 at the patient’s request. In May 2023, progression of the lung metastases was observed following a CT scan. Afatinib was resumed and PR was observed in July 2023. As of September 2023, the patient remains symptom free.

#### Case 4: delE709_T710insD

A 64-year-old female was diagnosed with NSCLC (stage IVB) in November 2017 during workup of a painful pathologic fracture in the 8th thoracic vertebra (**Figure 2B**). Radiologic imaging revealed a central tumor of the left lower lung lobe, as well as additional bone and brain metastases. Relevant comorbidities included arterial hypertension, chronic bronchitis, osteoporosis, and gastritis. NGS identified delE709_T710insD with a co-occurring *CD274/PD-L1* mutation (P146R).

The patient received whole-brain radiotherapy from November to December 2017, with palliative radiotherapy (thoracic spine, lumbar, 20 Gy, 5 Gy/fraction) in November 2017. The patient began first-line erlotinib (150 mg QD) plus intravenous zoledronic acid (4 mg every 3 weeks) in December 2017. Following PD in May 2018, treatment was discontinued in June. The patient received second-line afatinib (40 mg QD) starting in June 2018, and achieved a PR in July 2018. Subsequently, the patient experienced pneumonitis probably related to preceding radiotherapy of the thoracic spine, leading to death in September 2018 after approximately 3 months on treatment.

### Rare substitution mutations

#### Case 5: H988R substitution

**Figure 3:**
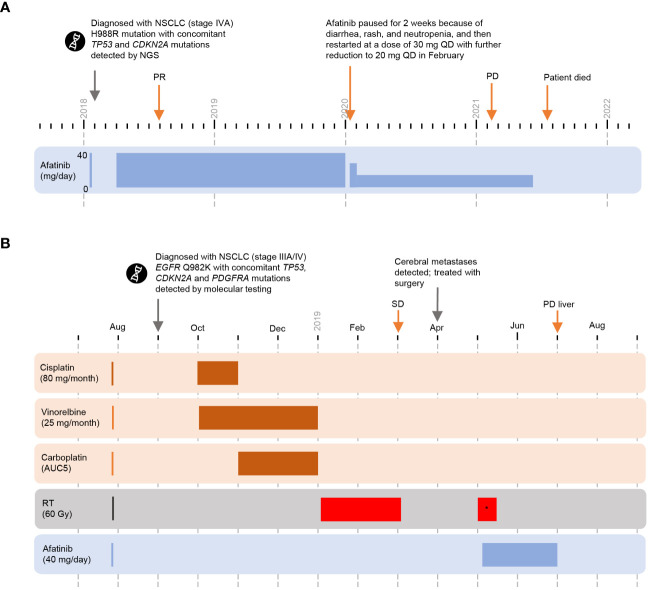
Cases 5 and 6 (novel substitution mutations) **(A)** Timeline of Case 5. **(B)** Timeline of Case 6. *whole brain radiotherapy. *CDKN2A*, cyclin-dependent kinase inhibitor 2A; *EGFR*, epidermal growth factor receptor; NGS, next-generation sequencing; NSCLC, non-small cell lung cancer; PD, progressive disease; PR, partial response; QD, each day; RT, radiotherapy; SD, stable disease; *TP53*, tumor protein 53.

A 74-year-old male presenting with weight loss was diagnosed with NSCLC (stage IVA, with pulmonary and pleural metastases) in February 2018 (**Figure 3A**). Relevant comorbidities included arterial hypertension, non-erosive reflux disease, chronic hepatitis C infection, hiatal hernia, and other gastrointestinal conditions. NGS testing confirmed a rare *EGFR* exon 25 mutation, H988R, with co-occurring *TP53* and *CDKN2A* mutations.

First-line treatment with afatinib (40 mg QD) started April 2018 and a PR was reported in August 2018. In January 2020, afatinib treatment was paused for 2 weeks because of diarrhea, rash, and neutropenia, and then restarted at a dose of 30 mg QD. The dose of afatinib was further reduced to 20 mg QD in February 2020. PR was maintained until at least February 2021 (date of last imaging). The patient deteriorated clinically without tumor progression and died in July 2021. Total duration on afatinib was 39 months; no other treatment was reported.

#### Case 6: Q982K substitution

In September 2018, an asymptomatic 65-year-old male with a history of arterial hypertension, latent diabetes mellitus, and degenerative spinal syndrome, was diagnosed with NSCLC (stage IIIA/IV) following magnetic resonance imaging examination of the cervical spine (**Figure 3B**); suspicious enlargement of the left adrenal gland was also observed. Positron emission tomography-CT standardized uptake values were 11–15 for the primary lung tumor and mediastinal lymph nodes, and four for the left adrenal gland. After discussion with the interdisciplinary tumor board, it was agreed to treat the patient according to stage III disease management practice and continue to monitor the left adrenal gland with serial imaging. Molecular testing confirmed a novel point mutation in *EGFR* exon 24 (Q982K) with co-occurring *TP53*, *CDKN2A*, and *PDGFRA* mutations.

Cisplatin (80 mg/m^2^ day 1 every 3 weeks) plus vinorelbine (25 mg/m^2^ day 1 and day 8 every 3 weeks), was initiated in October 2018. An episode of tinnitus prompted a switch from cisplatin to carboplatin (AUC 5) from cycle 2. Sequential radiotherapy (60 Gy) began in January 2019. Best overall response to chemoradiotherapy was SD in March 2019.

Cerebellar metastases were detected in April 2019, and were resected in the same month, followed by whole-brain radiotherapy in May 2019. The patient received second-line afatinib (40 mg QD) starting in May 2019. Despite a stable thoracic tumor, new liver lesions were detected in July 2019 and afatinib treatment was terminated. The total duration of afatinib treatment was 2 months.

### EGFR fusion

#### Case 7: *EGFR::CCDC6* fusion

**Figure 4:**
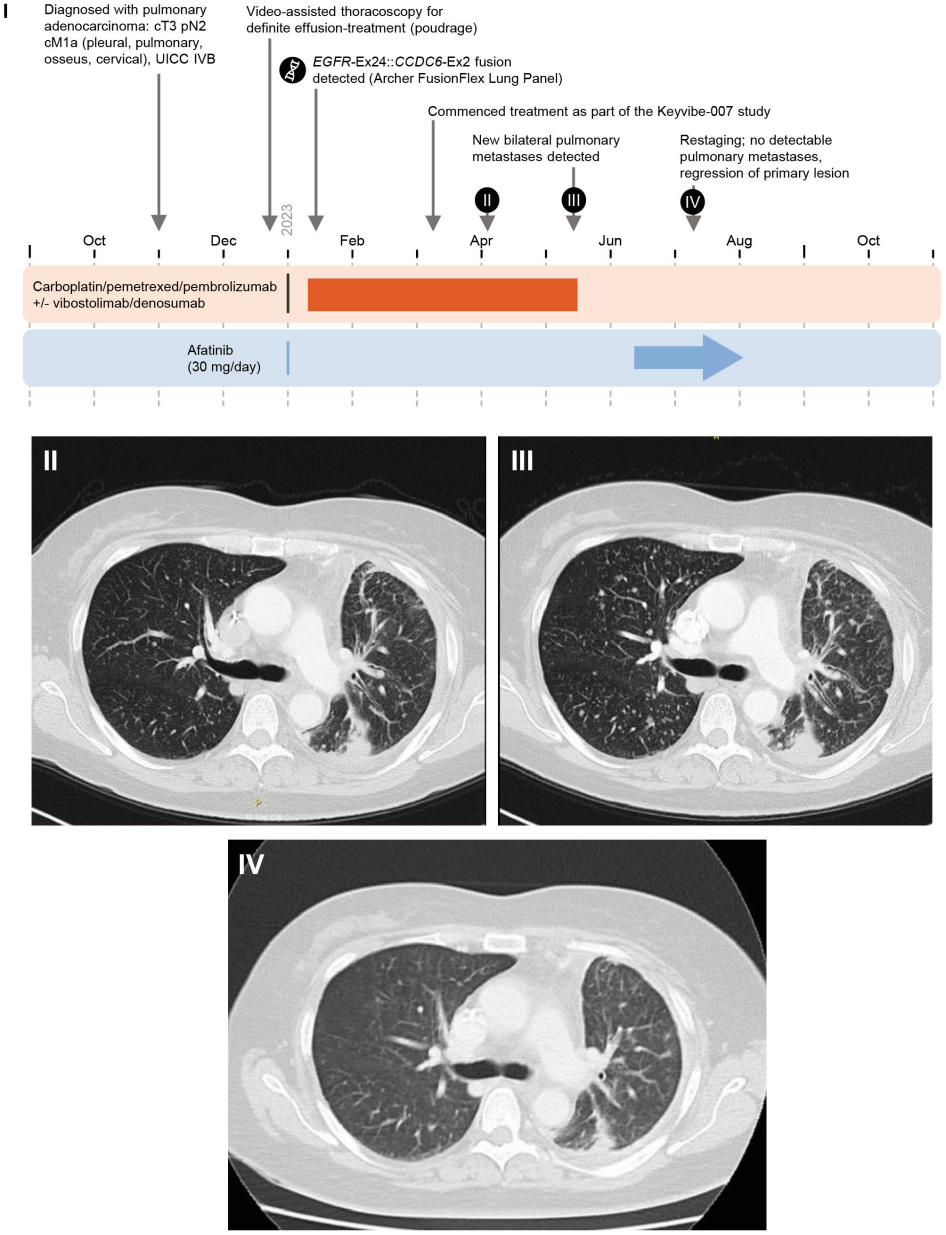
Case 7 (novel *EGFR::CCDC6* fusion) I. Timeline of Case 1. II. April 2023. III. May 2023, New bilateral pulmonary metastases detected. IV. July 2023. Restaging: no detectable pulmonary metastases, regression of primary lesion. Ex, Exon; UICC, Union for International Cancer Control.

A 56-year-old female ex-smoker (until 2005, 20 pack-years) with a history of bronchial asthma who presented with pleural effusion affecting the left thorax was diagnosed with stage IVB (UICC) adenocarcinoma NSCLC (primary lesion left lower lobe) in November 2022 (TTF-1-positive, *CK7* positive, Ki-67 score: 70%; PD-L1 Tumor Proportion Score 0%, PD-L1 immune cells 0%; cT3 pN2 cM1a [pleural, pulmonary, osseus, and cervical lymph nodes]). First-line treatment (carboplatin/pemetrexed/pembrolizumab ± vibostolimab/denusomab) as part of the KeyVibe-007 trial (EUDRA-CT: 2021-004564-94) began in January 2023 (**Figure 4**). At restaging in May 2023, multiple new bilateral pulmonary metastases were detected and participation in the trial ended.

Molecular testing (Archer FusionPlex Lung Panel) in January 2023 detected an *EGFR* exon 24::*CCDC6* (coiled-coil domain containing 6) exon 2 fusion. Treatment with second-line afatinib 30 mg QD began in June 2023. Regression of the primary lesion and complete resolution of pulmonary metastases were observed after 4 weeks. Treatment and response are ongoing.

## Discussion

This report describes outcomes with afatinib in NSCLC with a diverse range of extremely rare *EGFR* alterations found in routine clinical practice (**Supplementary Figure 1**). Five patients harbored rare aberrations that, to the best of our knowledge, have not been previously described in literature. Four patients had known PACC mutations either in isolation or as part of a compound *EGFR* mutation. Two patients had PACC mutations with co-occurring mutations in *TP53* or *PDGFRA*. One patient had an *EGFR* gene fusion, a rare type of driver event. Overall, these patients responded well to afatinib (**Supplementary Table 1**), consistent with preclinical modelling (4) and previous studies of afatinib treatment in patients with uncommon mutations (11, 28).

Cases 1 and 2 involved compound mutations comprising PACC and classical-like mutations which both responded to afatinib. Case 1 had a durable response to afatinib despite the presence of a co-occurring *TP53* mutation plus a novel compound *EGFR* mutation, that included substitution mutations on exons 18 (G719A; a PACC mutation) and 21 (L833F; a classical-like mutation). Cases 3 and 4 exhibited PACC exon 18 deletion insertion mutations, and both patients had a clinical response to afatinib treatment, including a long (>2 years) response reported for Case 3. Case 4 also harbored a concomitant *CD274/PD-L1* mutation, which we believe has not previously been described. Accumulating evidence indicates that delE709_T710insD is sensitive to afatinib and does not appear to be affected by the concomitant mutation. Cases 5 and 6 had *EGFR* substitution mutations in exon 25 (H988R) and exon 24 (Q982K), respectively; situated in the cytoplasmic region C-terminal domain, beyond the tyrosine kinase domain. Mutations here may destabilize receptor conformation, potentially causing upregulation of kinase activity and irregular downstream signaling (29). Both had co-occurring mutations in other genes, which are known to be prognostic biomarkers (23, 30–32). In Cases 5 and 6, prolonged survival was observed with first-line afatinib. In Case 7, the patient with the fusion, a dramatic response was observed in response to second-line afatinib. It is currently unknown how these rare mutations, and the *EGFR*::*CCDC6* fusion, align with the structure-based classification system (4), highlighting the difficulty associated with making treatment decisions for patients with novel mutations.

The selection of optimal treatment for patients with rare or compound *EGFR* mutations is often complex. Previous case reports describe compound mutations comprising substitution mutations classified as PACC and classical-like that respond to TKIs (33, 34). We found only one other report of a L833F-containing mutation, in which a patient with an L833F/L861Q mutation also achieved durable PR in response to first-line afatinib (progression-free survival [PFS]: 10 months) and clinical benefit to later-line osimertinib (34). A previous review briefly mentions the patient described in Case 5 with the H988R substitution (35). The review also mentions an additional patient with an H988R mutation who did not respond to afatinib treatment (35). While the recent structure-based classification system (4) has provided helpful information regarding predicted sensitivities of uncommon mutations, the sensitivities of rare compound mutations and the influence of co-occurring mutations remain difficult to predict in the absence of prospective clinical trials. Treatment decisions for these patients requires careful consideration.

Previous case reports of *EGFR* PACC insertion deletion mutations in NSCLC indicate sensitivity to afatinib and other EGFR TKIs (gefitinib, erlotinib) (36–38), including a 23-month PFS response with afatinib (37). Case 4 achieved a PR with afatinib after PD on erlotinib, which is also consistent with a previous case study where clinical benefit with afatinib following prior erlotinib treatment was reported (39). We have identified 16 reports of patients with a delE709_T710insD mutation who received EGFR TKIs (16), and consistent with the preclinical modelling (4), delE709_T710insD-mutated NSCLC appears to be more sensitive to afatinib than first-generation TKIs. In a review of 14 cases, PFS was significantly improved with afatinib compared with first-generation TKIs (median 7.0 *vs*. 3.1 months; *p* = 0.005) and all patients receiving afatinib achieved a PR (36).

Although *EGFR* gene fusions are rare, clinical responses in *EGFR* fusion-driven tumors have been reported with EGFR TKIs (40, 41). The *EGFR*::*CCDC6* fusion is novel, to our knowledge; however *CCDC6*-tyrosine kinase fusions (for example with *ALK*, *ROS1*, or *RET*), are recognized–and druggable–driver events in lung cancer (42). The durable response in this patient reinforces the importance of testing for fusion driver events, as this important class of somatic alteration can underly disease sensitive to targeted agents.

The interplay between *EGFR* alterations and co-occurring mutations in different genes represents a new frontier for NSCLC clinical research. In our case series, three patients had co-mutations in *TP53*, two had co-mutations in *CDKN2A*, and one had overexpression of PD-L1, plus a co-mutation affecting, *CD274/PD-L1*. These alterations occur commonly in patients with *EGFR*m+ NSCLC and have been associated with poor prognosis and resistance to TKIs (23, 30–32). However, in our case series, co-mutations did not prevent patients gaining benefit from afatinib treatment. Two patients with co-occurring *TP53* mutations exhibited prolonged time on treatment (35 and 39 months), durable response to afatinib was observed in one of the two patients with *CDKN2A* mutations (both patients with substitution mutations), and a PR was observed in the patient with a *CD274/PD-L1* mutation. Decisions about the initial treatment of NSCLC with uncommon *EGFR* mutations have key importance for the subsequent course and should be made carefully based on published evidence about TKI efficacy, as this can vary widely according to the specific mutation (43), and real-world data indicate that approximately 30–35% of patients do not receive treatment after the first line (44).

Reports of NSCLC with TKI-sensitive uncommon *EGFR* mutations may also prompt further clinical trial research in this setting; however, if clinical practice is to evolve, obstacles in terms of mutation testing must also be overcome. Current guidelines recommend that broad molecular profiling should be carried out for all patients diagnosed with NSCLC, which generally means NGS-based testing (9, 45). Globally, rates of molecular profiling in lung cancer patients are below 50% with a wide regional variation (46); financial constraints, quality and standardization of testing, access to testing, awareness, and turnaround times have all been cited as barriers to testing. Furthermore, some commonly used testing panels may miss uncommon mutations or those occurring outside of Exons 19, 20, and 21 (47, 48). Advances in testing strategies and methodologies have the potential to improve molecular profiling in NSCLC; these include the adoption of the structure-based classification system into testing panels and the use of liquid biopsy as a rapid, non-invasive means of assaying genomic profiles (49). Liquid biopsy may be particularly useful for monitoring temporal changes in mutation and biomarker status and is already in use to detect resistance to EGFR TKIs (50).

Integration of classification systems and real-world evidence may support future treatment decisions in patients with uncommon *EGFR*m+ NSCLC. Better understanding of the impact of co-occurring mutations is required. Patients with uncommon *EGFR* mutations are now being included in several ongoing randomized clinical trials assessing the efficacy and safety of EGFR TKIs in NSCLC (51–54). In a recent analysis of the phase III ACHILLES trial in treatment-naive patients with uncommon or compound *EGFR* mutations, PFS with afatinib (10.6 months) was significantly longer than with platinum-based chemotherapy (5.7 months; hazard ratio: 0.422, *p* = 0.0007), supporting the use of first-line EGFR TKIs in this setting (55).

## Conclusion

These cases corroborate the clinical and preclinical evidence that certain uncommon *EGFR* mutations are sensitive to afatinib. This series illustrates the importance of further study in this area and the need for publicly available mutation databases to support prescribing decisions in the absence of prospective clinical trial data for patients with rare mutations.

## Data availability statement

The datasets generated and analyzed during the study are available from author AS on reasonable request. Requests to access these datasets should be directed to AS: anja.stammberger@boehringer-ingelheim.com.

## Ethics statement

Ethical approval was not required for the studies involving humans because Manuscript details a collection of case studies from patients treated in routine practice, not results for a formal study. The studies were conducted in accordance with the local legislation and institutional requirements. The participants provided their written informed consent to participate in this study. Written informed consent was obtained from the individual(s) for the publication of any potentially identifiable images or data included in this article. Written informed consent was obtained from the participant/patient(s) for the publication of this case report.

## Author contributions

PC: Data curation, Formal analysis, Writing – review & editing. FH: Data curation, Formal analysis, Writing – review & editing. PH: Data curation, Formal analysis, Writing – review & editing. MF: Data curation, Formal analysis, Writing – review & editing. MT: Data curation, Formal analysis, Writing – review & editing. H-GK: Data curation, Formal analysis, Writing – review & editing. AA: Data curation, Formal analysis, Writing – review & editing. AS: Data curation, Formal analysis, Writing – review & editing. EL: Data curation, Formal analysis, Writing – review & editing.
