# Supplementary material for: Activity of afatinib in patients with NSCLC harboring novel uncommon EGFR mutations with or without co-mutations: a case report
Source: Front Oncol. 2024 May 6;14:1347742. doi: 10.3389/fonc.2024.1347742 (PMC11103604; doi:10.3389/fonc.2024.1347742)
Supplement: Supplementary file 1 [file DataSheet_1.docx]

# SUPPLEMENTARY TABLE 1 Overview of mutations and clinical responses to afatinib.

| Patient | *EGFR* mutation | Concomitant aberrations | Line of treatment with afatinib | Time on afatinib treatment, months | Best response |
| --- | --- | --- | --- | --- | --- |
| **1** | G719A+ L833F | *TP53* p.T140fs | 1L | 35 | PR |
| **2** | G719A+ L861R | PD-L1: 1% | 1L | 5 | PR |
| **3** | delE709_T710insD | None reported | 1L | >36 (ongoing) | PR (resolution of symptoms) |
| **4** | delE709_T710insD | *CD274:* allele frequency 43% in Ex4, P146R | 2L | 3 | PR |
| **5** | H988R | *CDKN2A*:  Ex1, p. W15, allele frequency 20%; *TP53*:  Ex8, V272L, allele frequency 29% | 1L | 39 | PR |
| **6** | Q982K | *TP53*:  Ex7, S261I, allele frequency 60.9%;  *CDKN2A*:  Ex2, G116A, allele frequency 55.9%; *PDGFRA:*  Ex11, L521M, allele frequency 46.1% | 2L | 2 | PD |
| **7** | *EGFR* (Exon 24)::*CCDC6 (*Exon 2) Fusion | None reported | 2L | >2 (ongoing) | PR (regression of primary lesion; complete resolution of pulmonary metastases) |

1/2L, first/second-line; *EGFR*, epidermal growth factor receptor; Ex, exon; fs, frame shift; PR, partial response; p., point mutation; PD, progressive disease.

#
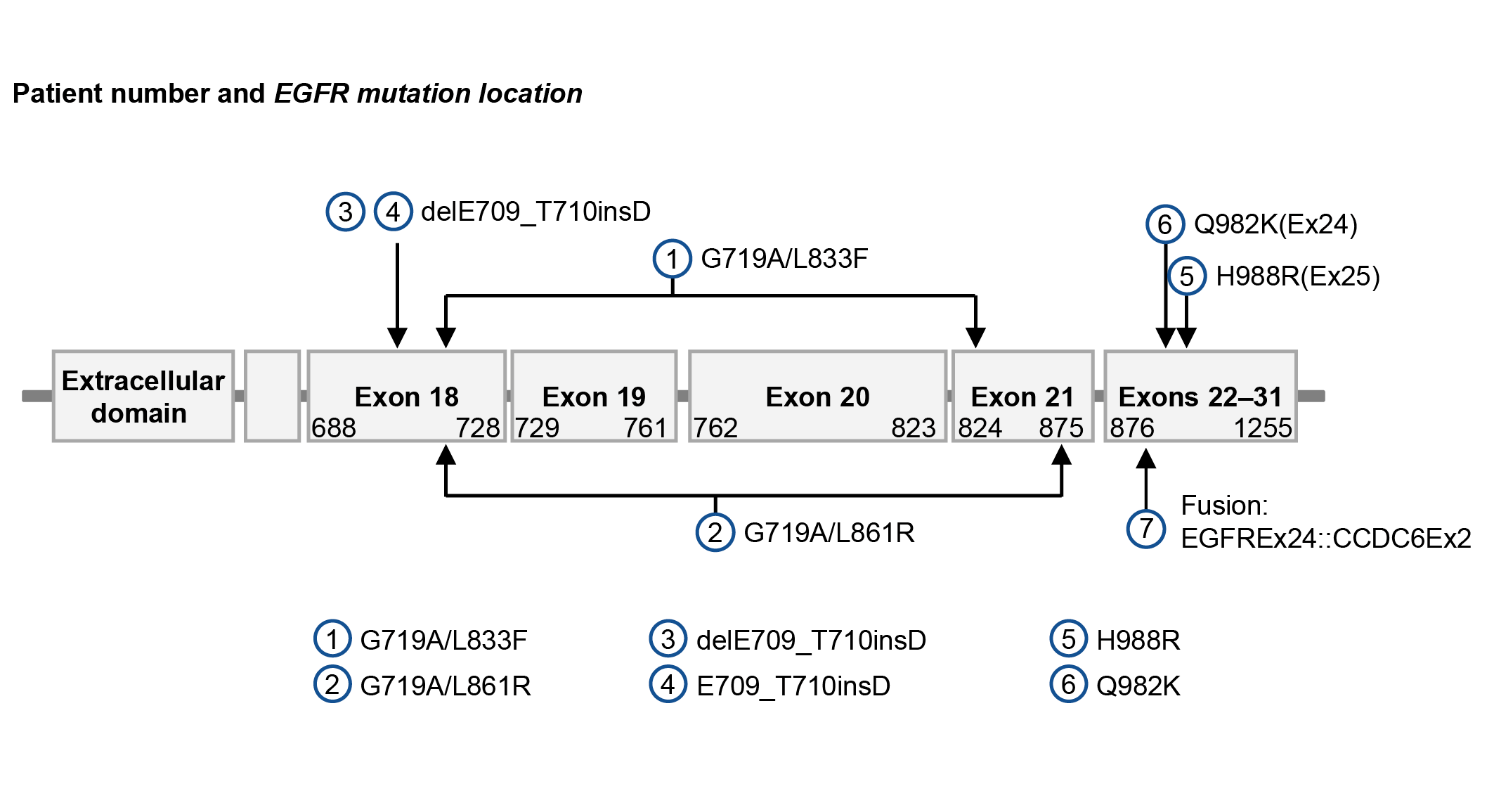


**SUPPLEMENTARY FIGURE 1**
Schematic of mutation locations on the *EGFR* gene. Circled values indicate patient number. Figure based on Robichaux 2021 (1). *EGFR*, epidermal

1. Robichaux JP, Le X, Vijayan RSK, Hicks JK, Heeke S, Elamin YY, et al. Structure-based classification predicts drug response in EGFR-mutant NSCLC. *Nature* (2021) 597(7878):732–7. doi: 10.1038/s41586-021-03898-1
